# A hepatic sclerosing hemangioma emerged in the postoperative course of multiple gastric carcinoid tumors masquerading as metachronous liver metastasis

**DOI:** 10.1016/j.ijscr.2019.03.018

**Published:** 2019-04-04

**Authors:** Ryota Koyama, Nozomi Minagawa, Yoshiaki Maeda, Toshiki Shinohara, Tomonori Hamada

**Affiliations:** Department of Gastrointestinal Surgery, Hokkaido Cancer Center, Sapporo, Japan

**Keywords:** Hepatic sclerosing hemangioma, Gastric carcinoid, Metachronous liver metastasis

## Abstract

**Introduction:**

Hepatic sclerosing hemangiomas are rare tumors whose appearance on imaging is similar to that of malignant tumors. The resulting difficulties in preoperative diagnosis frequently lead to surgical resection in order to accurately identify the tumor.

**Case presentation:**

A 68-year-old man was diagnosed with multiple gastric carcinoid tumors (T1N1M0, pStage IIIB) after total gastrectomy with D2 lymph node dissection. Enhanced computed tomography of the abdomen 2 years after the initial surgery revealed a novel solid lesion with ring enhancement measuring 22 × 15 mm at S5 of the liver. Metachronous liver metastasis from multiple gastric carcinoid tumors was suspected, and partial hepatectomy of S5 was successfully performed. Histopathological diagnosis was sclerosing hemangioma with no malignant findings.

**Discussion:**

Ours is the first reported case of a newly appearing sclerosing hemangioma excised and diagnosed histopathologically. Hepatic sclerosing hemangiomas are rarely observed, but they are often resected under a preoperative diagnosis of malignant lesion due to the similarity on imaging studies.

**Conclusion:**

Surgeons must consider that a hepatic tumor may be a sclerosing hemangioma, particularly when the lesion appears to be ring enhanced.

## Highlights

• The authors present an unusual case of newly appearing hepatic sclerosing hemangioma newly emerged in the postoperative course after the resection of multiple gastric carcinoid tumors.
• Hepatic sclerosing hemangioma is often misdiagnosed as malignant lesion for its resemblance on the imaging studies.
• Although preoperative diagnosis is still difficult, hepatic sclerosing hemangioma must be included into the differential diagnosis of hepatic tumors.

## 1 Introduction

Hepatic hemangiomas are common lesions observed in the general population. They are rarely resected because of the characteristics on imaging studies. However, these lesions may alter into hepatic sclerosing hemangiomas when the hemangioma is denatured and replaced by connective tissue [1]. On imaging, hepatic sclerosing hemangiomas resemble malignant lesions, thereby leading to the resection of the tumor due to the differential diagnosis of malignancy. Previous reports have noted that the size of a hepatic hemangioma alters during the time course due to intratumoral bleeding [2]. However, there have been no reports of newly appearing tumors that increase in size over a short period of time. In this report, we discuss a case of a hepatic sclerosing hemangioma resected under the preoperative diagnosis of metachronous liver metastasis from multiple gastric carcinoid tumors. We further report and review previous studies on these lesions. This work has been reported in line with the SCARE criteria [3].

## 2 Presentation of case

**Fig. 1:**
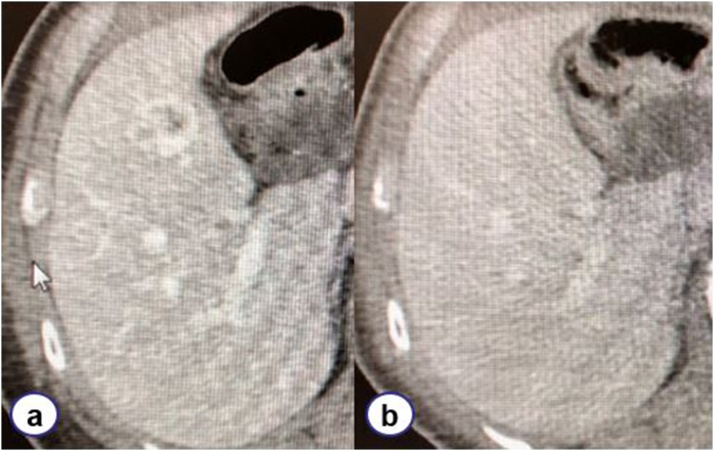
Computed tomography imaging. On computed tomography imaging conducted 2 years postoperatively, a mass with ring enhancement measuring 22 × 15 mm in S5 emerged (a) compared to the scan performed 1 year postoperatively (b).

**Fig. 2:**
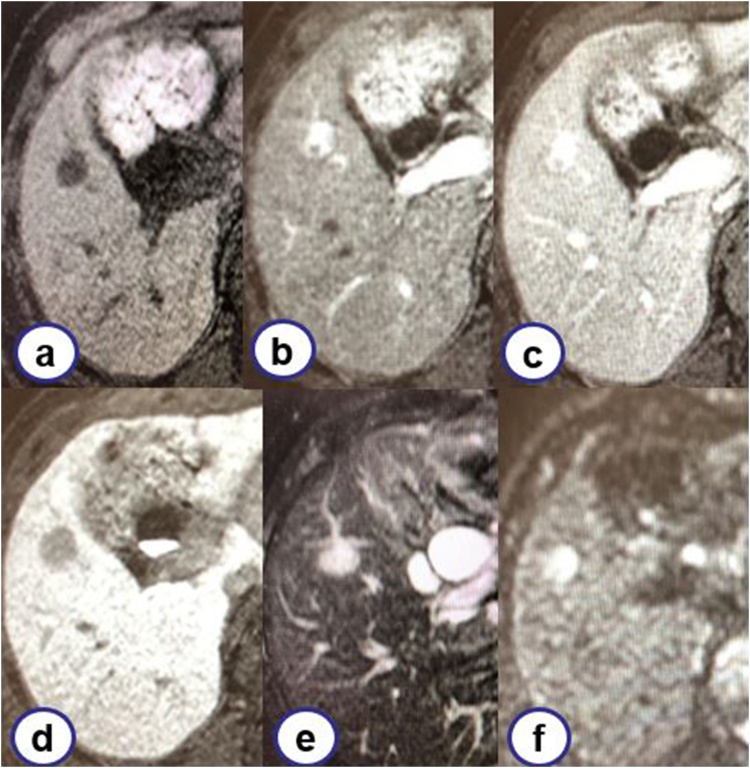
Gadolinium ethoxybenzyl diethylenetriamine pentaacetic acid-enhanced magnetic resonance imaging. A mass showing low intensity and measuring 17 mm was noted on T1-weighted image (a). The dynamic study showed gradual enhancement (b: 30 s. c: 180 s. d: hepatocyte phase). The mass showed high intensity on T2-weighted image (e) and diffusion-weighted image (f).

**Fig. 3:**
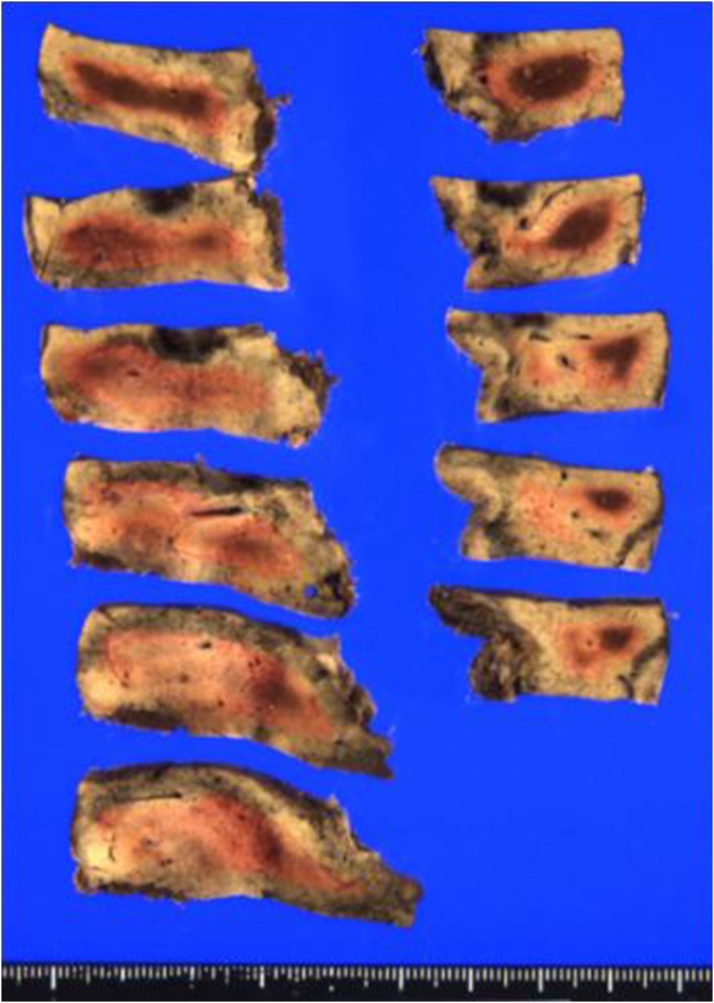
Macroscopic findings. A well-marginated nodular lesion with mixture of whitish and reddish region measuring 15 × 11 × 19 mm was noted.

A 68-year-old man underwent total gastrectomy with D2 lymph node dissection and cholecystectomy 2 years ago under the diagnosis of gastric carcinoid tumors. The pathological diagnosis was multiple gastric carcinoid tumors with lymph node metastasis (T1N1M0, pStage IIIB) and normal gall bladder (Fig. 1). The patient was regularly followed up with laboratory tests and imaging studies with no signs of recurrence. After 2 years, enhanced abdominal computed tomography (CT) revealed a novel lesion in S5 of the liver with ring enhancement measuring 22 × 15 mm (Fig. 2). The diagnosis of metachronous solitary liver metastasis from multiple gastric carcinoid tumors was made. Because there was no sign of recurrence in the other organs, we decided to surgically resect the lesion. The patient was 158 cm in height and weighed 48 kg (BMI, 19.2). The abdomen was soft and flat with the surgical incisional scar in the upper region. Laboratory data showed elevated transaminases (AST, 143 IU/l; ALT, 180 IU/l) and slight anemia (hemoglobin, 12.1 g/dl). Other values, including tumor markers (carcinoembryonic antigen and carbohydrate antigen 19-9), were within normal limits. Enhanced abdominal CT revealed a ring-enhanced mass that had been undetectable on the site by the CT conducted 12 months ago. Other findings were liver cysts in S3 and S4, both of which remained unchanged. The hepatocyte phase of the gadolinium ethoxybenzyl diethylenetriamine pentaacetic acid-enhanced magnetic resonance imaging (MRI) showed a low-intensity mass measuring 17 mm in S5 (Fig. 3); the mass showed high intensity on T2-weighted image and diffusion-weighted image. The enhanced dynamic study revealed gradually enhanced effect with ringed enhancement. Based on these findings, a diagnosis of a metachronous solitary metastasis from multiple gastric carcinoid tumors was made, and we decided to resect the lesion by laparotomy. The liver was evaluated as A (5 points) according to Child–Pugh classification.

**Fig. 4:**
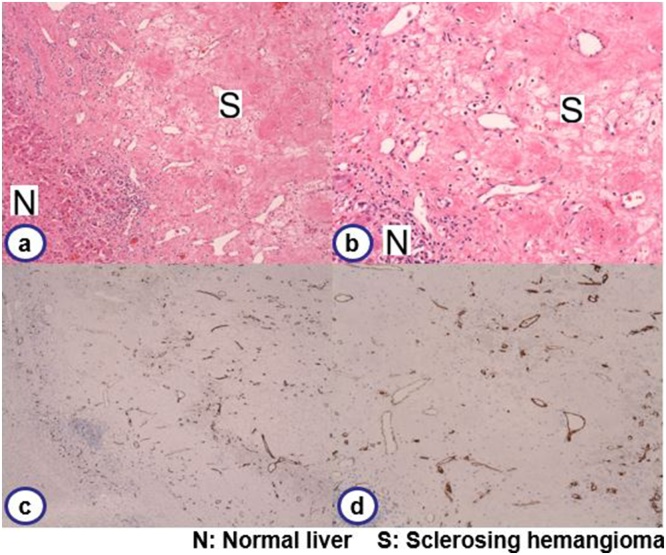
Histopathological findings. A fibrous lesion with collagenous stroma replacing hepatocytes was noted. Lack of cellular component and edematous tissue with micro-bleeding was observed. Dilated vessels were dispersed in the lesion (a: HE. × 20. b: HE. × 100). Immunohistochemistry showed CD34 positive endothelium, indicating dilated vessels in the lesion (c: HE. × 20. d: ×100).

Laparotomy was performed, and we confirmed that no other metastatic lesion was present by intraoperative exploration using ultrasonography. Following the ultrasonography-guided marking on the liver surface, S5 partial hepatectomy was successfully performed under total hepatic ischemia with the Pringle maneuver. The total ischemic time was 135 min, the operation time was 306 min, and the bleeding was 1320 ml. The resected specimen showed a 15 × 11 × 19 mm well-marginated nodular lesion with a mixture of whitish and reddish regions (Fig. 3). Histopathological findings revealed a fibrous nodular lesion without hepatocytes (Fig. 4). Scarce cellular components and micro-bleeding were also observed. Dilated vessels were dispersed inside the lesion, leading to the diagnosis of denatured hemangioma, i.e., hepatic sclerosing hemangioma.

The postoperative course was uneventful, and the patient was discharged on postoperative day 19.

## 3 Discussion

Hepatic hemangiomas are frequently observed benign lesions among the tumorous lesions occurring in the liver. They are classified into capillary hemangiomas, cavernous hemangiomas, and sclerosing hemangiomas [4]. Small capillary hemangiomas are of no clinical importance. Larger cavernous hemangiomas sometimes require surgical attention when they cause symptoms, particularly giant hemangiomas, which can cause coagulopathy [5] or spontaneous rupture [6]. It is important to note that there has been no report of malignant transformation of liver hemangiomas to date. The characteristic imaging study finding is of a space occupying lesion with rapid flow that has peripheral enhancement progressing centripetally [7]. Due to this distinct characteristic, the differential diagnosis is rather easy. However, the third sclerosing hemangioma sometimes requires differential diagnosis with malignant tumors due to its resemblance to such tumors on imaging. The histopathological characteristic is the dense fibrous tissue after the secondary degeneration by involution or thrombosis [8]. The imaging study is the key modality for differential diagnosis of liver masses. Cavernous hemangiomas have rapid enhancement expanding centripetally and showing high density on the equilibrium phase. These lesions show well-circumscribed high intensity on T2-weighted imaging by MRI. Conversely, sclerosing hemangiomas are poorly enhanced on the equilibrium phase on enhanced CT, and MRI shows heterogenous intensity [7]. In a recent report summarizing 32 cases of hepatic sclerosing hemangiomas, these tumors were mentioned to appear mainly as low density on plain CT and ring enhancement on enhanced CT. MRI showed low intensity on the T1-weighted imaging and high intensity on T2-weighted imaging. Moreover, preoperative diagnosis included mostly hepatic metastases, hepatocellular carcinomas, and biliary carcinomas [9]. Interestingly, hepatic sclerosing hemangiomas have a propensity to degenerate with fibrosis starting from the center of the lesion and extending centrifugally, suggesting formation by degenerative alteration from ischemia [7]. The etiology of liver hemangiomas is considered to be partially familial [4]. It is reported that hepatic hemangiomas are observed in 5%–10% of 1-year-old infants. Most of these tumors degenerate and disappear, although some remain and grow larger over time [4]. Given these findings, in our case, we assert that a small undetectable hemangioma existed preoperatively and became larger due to trauma from surgeries or other causes.

Generally, follow-up or surgical resection is the management techniques of choice for hepatic hemangiomas. Sakamoto et al. analyzed 510 cases of surgical resection of hepatic hemangiomas and stated that surgical indication can be stratified according to the diameter of the hemangioma [10]. First, a mass under 5 cm has no characteristics because it is too small to appear in the typical image, and of tumors under 5 cm, 43.5% were suspected as malignancy preoperatively. Second, for asymptomatic masses of 5–10 cm, follow-up is usually enough because the pretest probability is high enough to exclude any other diagnosis. Finally, for masses >10 cm and with abdominal symptoms or coagulopathy, surgical resection is indicated. If there is small percentage of doubt, surgical resection should be performed.

The most frequent distant metastatic organ from gastric carcinoid is liver. Treatment strategy depends on the intrahepatic distribution of the metastatic lesion. According to the ENETS Consensus Guideline, anatomical hepatectomy is recommended for the cases confined to one lobe or two adjacent segments [11]. For cases distributed in both lobes, combined surgery and ablation therapy is the recommendation. Diffuse lesion is inoperable. Histopathologically, NET G1 and G2 without extrahepatic lesion are candidates for surgery [12]. Chaoyong et al. reported that the 3-year survival rate of stage Ⅲ gastric carcinoid tumors was 51.1% [13]. Recurrence after 16 years of gastric carcinoid resection is also reported. Therefore, careful and long term follow-up is required for gastric carcinoid tumors [14].

In our case, we chose to resect the newly appeared liver mass during the postoperative follow-up after the multiple gastric carcinoid resections. To the best of our knowledge, there has been no such report previously. Hepatic sclerosing hemangiomas are difficult to diagnose because they shows findings similar to malignant lesions. Therefore, malignancy must be included in the differential diagnosis of hepatic lesions.

## Conflicts of interest

The authors (RK, NM, YM, TS & TH) declare no conflicts of interests or disclosures.

## Sources of funding

This work received no funding.

## Ethical approval

This study is exempt from ethical approval in our institution.

## Consent

Consent obtained.

## Author contribution

RK is the primary investigator and contributed to conceptualization, data collection and drafting the manuscript. NM, YM, TS, TH supervised and checked the manuscript. All authors have read and approved this manuscript for publication

## Registration of research studies

NA

## Guarantor

Ryota Koyama

Tomonori Hamada

## Provenance and peer review

Not commissioned, externally peer-reviewed
